# Bilateral absence of a lunate: a case report

**DOI:** 10.1080/23320885.2026.2756630

**Published:** 2026-11-06

**Authors:** Anja Imsirovic, Jorma Ryhänen, Panu H. Nordback

**Affiliations:** a Department of Plastic, Reconstructive and Aesthetic Surgery, Medical University of Innsbruck, Innsbruck, Austria; b Department of Hand Surgery, Bridge Hospital, Helsinki University Central Hospital and University of Helsinki, Helsinki, Finland

**Keywords:** Absent lunate, bilateral, isolated, pain, wrist

## Abstract

Absence of the lunate bone in adults is extremely rare, with few reported cases. Most cases are bilateral and congenital, without syndromic features; some are associated with other carpal anomalies, including absent triquetrum or hypoplastic scaphoid. We report this rare condition with an updated literature review and a suggested treatment algorithm. We conducted a structured literature review of published human cases of unilateral or bilateral lunate absence. Major databases were searched from 1946 until July 2025. We also describe a 51-year-old woman attending our outpatient clinic. Our search identified six articles describing this pathology. Lunate absence is predominantly bilateral, with only three unilateral cases reported. Our patient presented with bilateral wrist pain aggravated by loading and difficulty performing push-ups. Initial radiographs suggested an enlarged scapholunate gap and a deformed lunate fossa. Computed tomography confirmed bilateral lunate absence. Patient’s symptoms improved following night splinting and wrist stretching exercises. As symptoms remained tolerable with conservative treatment, surgery was not performed. Adult lunate absence is a rare congenital abnormality. Diagnosis requires thorough history-taking, clinical examination, and advanced imaging to identify associated abnormalities. Treatment should be individualized according to symptom severity and functional limitations. Conservative management includes activity modification, splinting, physiotherapy, and medication. For patients with persistent pain or significant functional impairment despite conservative treatment, surgical options, including partial or total wrist fusion, may be considered. This case highlights the importance of recognizing this uncommon condition and tailoring management to clinical presentation. Further research is needed to establish standardized diagnostic criteria and treatment recommendations.

## Introduction

Congenital abnormalities of the carpal bones are rare, accounting for a small proportion of upper limb developmental anomalies. These abnormalities may involve variations in the number, morphology, maturation, or fusion of carpal bones and can occur as isolated findings or as part of congenital syndromes. The carpal ossification process follows a predictable sequence, beginning with the capitate and hamate during infancy and extending to the lunate, scaphoid, and other carpal bones throughout childhood; therefore, the absence of a carpal bone after the expected period of ossification is an uncommon finding requiring careful evaluation [1–3].

The lunate bone plays an important role in carpal stability and load transmission between the forearm and distal carpal row. Absence of the lunate in adulthood is an exceptionally rare condition, with only a limited number of cases reported in the literature. Proposed explanations include congenital failure of ossification, delayed ossification, fusion with adjacent carpal bones, resorption after normal development, or destruction of the cartilaginous precursor during early life, including possible septic causes [4]. Although congenital carpal abnormalities may be associated with other skeletal anomalies or syndromic conditions, isolated lunate aplasia without associated abnormalities is particularly uncommon [3,5].

We present a rare case of bilateral congenital absence of the lunate bone in an adult patient with secondary degenerative changes, predominantly affecting the dominant wrist. This report aims to review the current literature, describe the clinical and radiological findings, discuss possible developmental mechanisms, and outline appropriate management strategies for this unusual condition. Written informed consent for publication was obtained from the patient. Ethical approval was not required for this case report, which was conducted in accordance with the principles of the Declaration of Helsinki.

## Patient

A 51-year-old right-handed female non-smoker office worker was referred in January 2024 for ­bilateral wrist pain. Her medical history included chronic ulcerative proctitis, menopausal hormone replacement therapy, carpal tunnel release 10 years earlier, and septic meningitis in infancy. She reported a decade-long history of gradually worsening wrist pain and stiffness, aggravated by desk work and exercise, with a small dorsal lump on the right wrist. The right wrist was more ­symptomatic, with activity-related bilateral numbness but minimal resting pain. She denied any trauma. Her sister had unspecified wrist problems, while her four children (aged 12–22 years) were asymptomatic. Examination revealed a mildly prominent right ulna; other findings are summarized in Table 1.

**Table 1.** Findings on clinical examination.

**Inspection**	Right/Left
Scar	None/Faint scar after carpal tunnel release
**Symptoms & Palpation**	Right/Left
Synovitis	None/none
Tenderness	Around lunate/ none
**Range of Motion**	Right/Left
Flexion	40°/50°
Extension	40°/45°
Pronation	80°/80°
Supination	70°/70°
**Strength & Sensation**	Right/Left
Grip Strength	31/30 kg
Sensation	Intact/intact
**Joint Stability & Special Tests**	Result
Distal Radioulnar Joint	Stable
Drawer Test	Negative
Lichtman Test	Negative
Ulnocarpal Stress Test	Unremarkable
Durkan’s Test	Positive (right)
Phalen’s Test	Negative bilaterally
Tinel’s Test	Negative bilaterally
**Patient-Reported Outcome (PRWHE)**	Right/Left
Pain Score	27/4
Function Score	11/0

Plain radiographs (Figure 1) showed an absent lunate with only a small residual ossification centre on the right, an underdeveloped lunate fossa, hypoplastic capitate, and degenerative changes of the right radiocarpal joint. CT (Figures 2 and 3) confirmed congenital failure of lunate ossification with a residual ossification centre.

**Figure 1.**
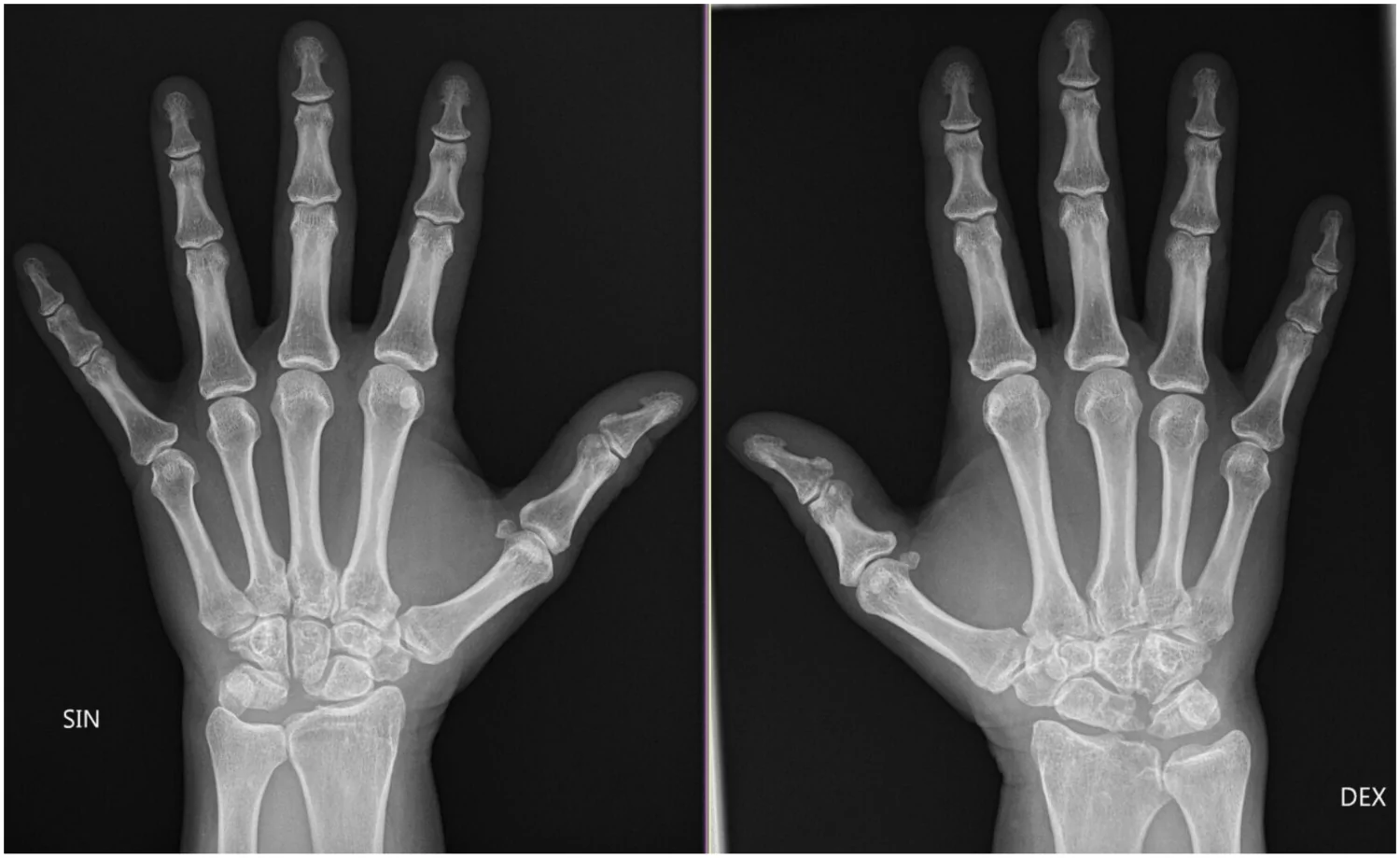
Bilateral wrist radiograph (AP view).

**Figure 2.**
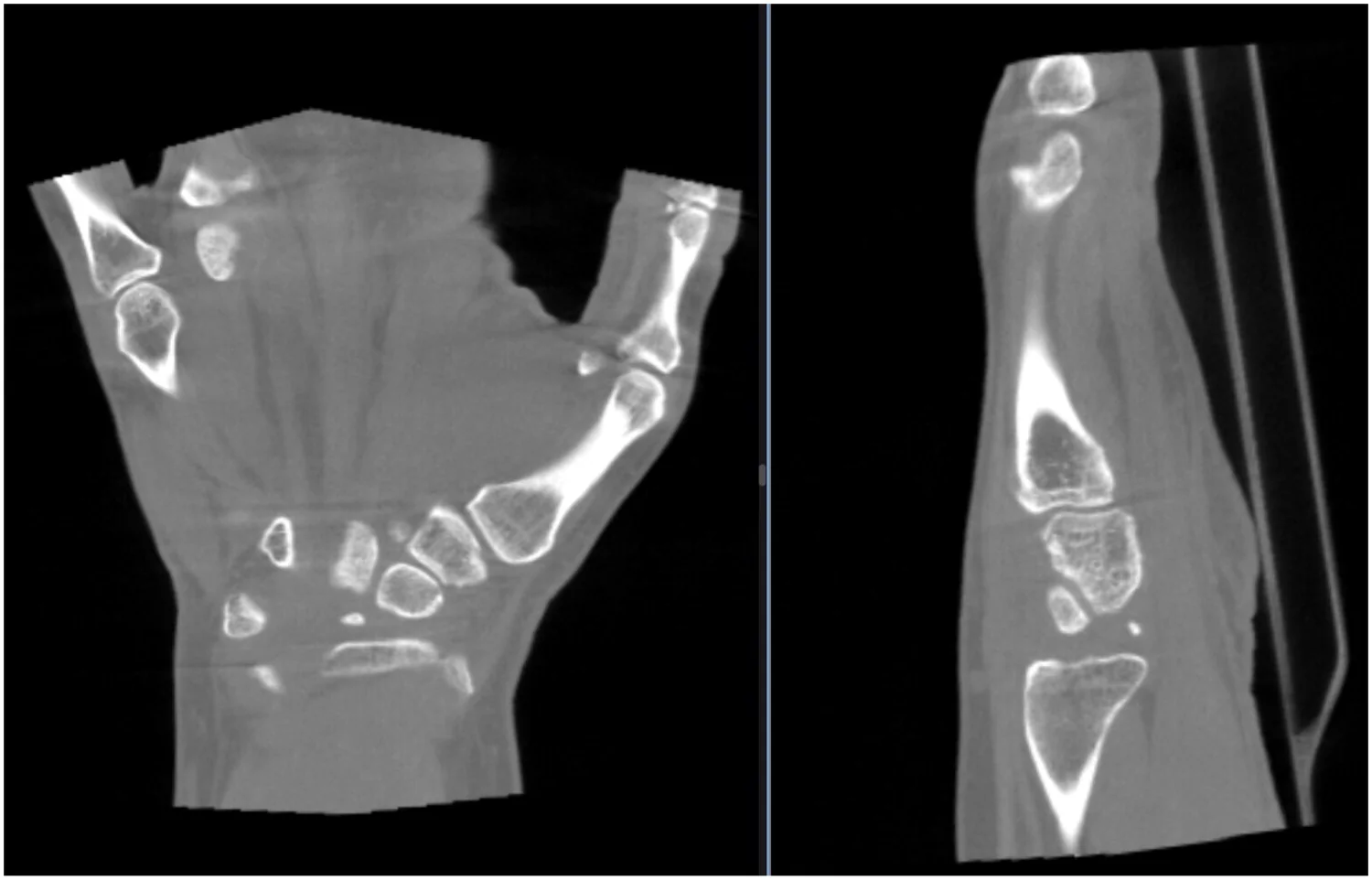
Left wrist CT imaging (coronal and sagital views).

**Figure 3.**
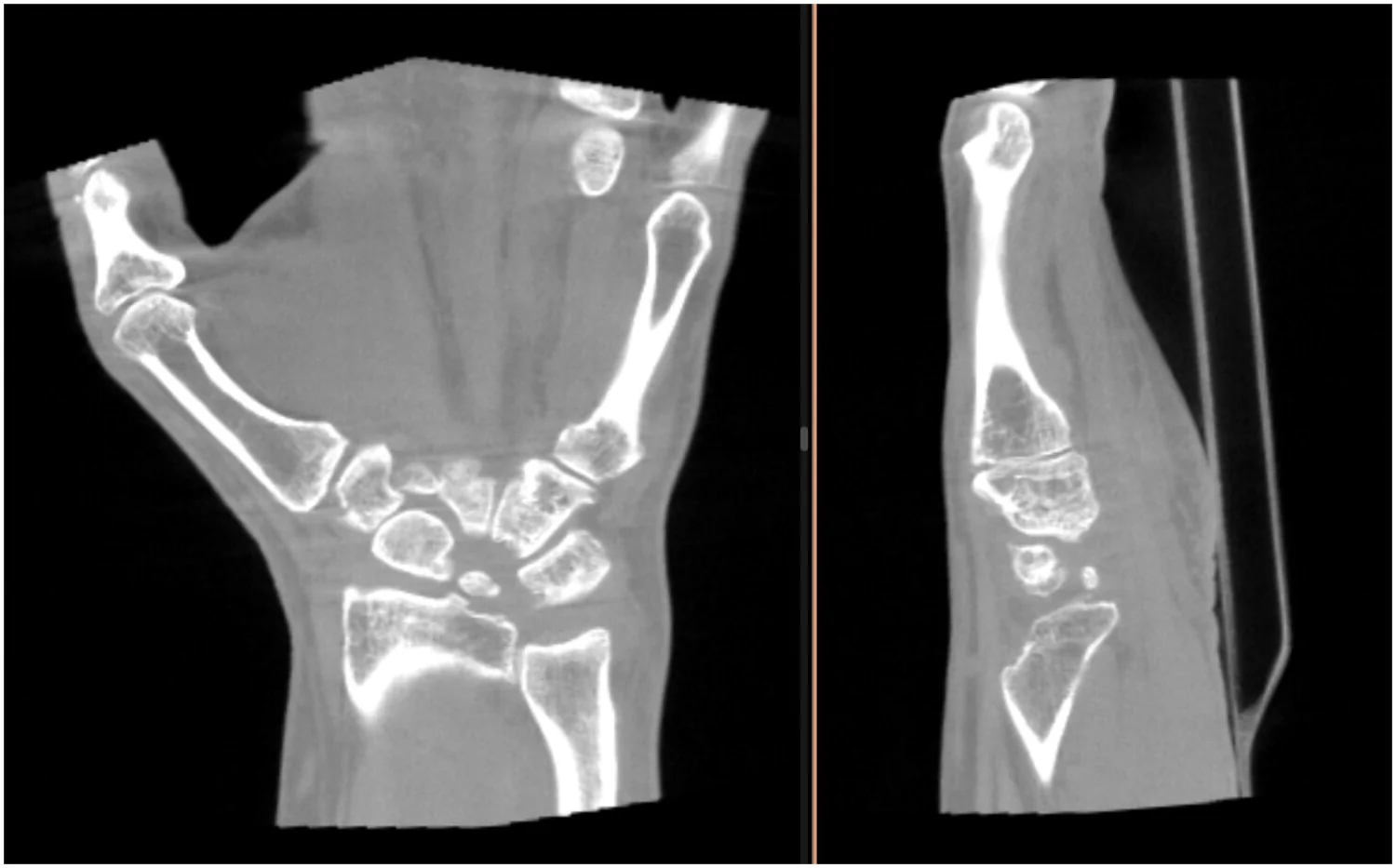
Right wrist CT imaging (coronal and saggital views).

A diagnosis of congenital absent lunate with secondary degenerative changes, more pronounced on the dominant right side, was made. Conservative treatment included splinting, analgesia, ergonomic modification, and possible corticosteroid injection. A neve conduction studies (ENMG) was arranged for intermittent finger numbness, and screening of her children was considered unnecessary unless symptoms developed.

At 4-month follow-up, splints had improved symptoms, although activity-related numbness and clicking persisted. ENMG was normal, excluding median and ulnar neuropathy. Physiotherapy was recommended, with surgery reserved for worsening pain or progression of arthritis.

During hand therapy, she reported continued benefit from night splints but persistent pain during activities such as cycling. She was instructed in stretching, soft-tissue release, self-massage, gradual weight-bearing using push-up handles, and progressive strengthening. Upon teleconsultation in one month, she reported further improvement and was advised to return if symptoms worsened.

## Literature review

We conducted a structured literature review to identify published articles on “absent lunate and unilateral or bilateral” in humans. Embase, MEDLINE, PubMed, Google Scholar, and Cochrane were searched from 1946 to July 2025, identifying six relevant articles (Table 2). Full-text review was performed, and data on study design, patient characteristics, anatomical findings, and outcomes were extracted.

**Table 2.** Characteristics of included studies.

Study (Author, year)	Patient (Male/female)	Age	Handiness	Anomaly (lunate, other)	Unilateral/ bilateral	Treatment	Other
Dwivedi et al. 2023	Male	20	N/A	Absent lunate and triquetrum	Unilateral right	NSAID, Muscle strengthening exercises	N/A
Edwin et al. 2024	Male	36	N/A	Absent lunate	Unilateral right	Conservative	Hypoplastic upper limb, 2 cm shorter than left side
Kobayashi, 1991	Male	20	N/A	Absent lunate	Bilateral	N/A	Presented with left wrist pain
Newcomb et al. 2021	Male	45	Right	Absent lunate, hypoplastic scaphoid	Unilateral left	Conservative	N/A
Postacchini et al. 1975	Male	38	N/A	Absent scaphoid, lunate and triquetrum	Unilateral right	Conservative	Wrist pain and limb shortening
Roche et al. 1967	Female	12	N/A	Absent lunate	Unilateral left	Conservative	Straph Septicemia at the age of 4
de Smet et al. 2005	N/A	N/A	N/A	Absent scaphoid and lunate	N/A	N/A	N/A

Published reports of carpal agenesis most commonly involve the scaphoid, either alone or with other carpal bones. Reported cases include absent lunate with hypoplastic scaphoid (Newcomb et al.), isolated absent lunate (Roche et al.), absent scaphoid, lunate, and triquetrum (Dwivedi et al.), bilateral absent lunate (Kobayashi et al.), and absent scaphoid with lunate (De Smet).

According to Roche et al. (1967), several explanations exist for an absent lunate. It may be congenitally absent, or its ossification may simply be delayed, sometimes becoming the seventh carpal bone to ossify and potentially slowed by illness during its early development. In other cases, the lunate may fuse with the capitate, or it may have ossified normally but later undergone resorption. Another possibility is that a septic process destroyed the lunate while it was still cartilaginous [4].

Furthermore, congenital absence of a carpal bone is typically associated with an anomaly of the radius or ulna. The first case of bilateral, isolated lunate bone deficiency was described by Kobayashi et al. in 1991 [5].

When it comes to isolated congenital aplasia of the carpal bones, in the absence of an associated congenital syndrome, is exceedingly rare. Newcomb et al. [6] describe a case involving the absence of the lunate bone and a dysplastic scaphoid bone. There was no suggestion of any remnant lunate bone, which would have led to the suspicion of Kienbock’s disease. To date, only two cases of unilaterally absent lunate have been reported in the literature [4,6]. The pathophysiological mechanisms behind carpal bone agenesis are unknown. The lunate usually completes ossification between the ages of 15 and 25. An additional proposed mechanism is idiopathic, non-hereditary osteolysis of the carpal chondrification centre in utero or during childhood [6]. In the most recent case report by Edwin et al. [7], a 36-year-old man presented with diffuse dorsal wrist pain. Radiographs revealed an absence of the lunate bone on the right side. However, a comparative X-ray of the contralateral side showed a normal carpus. In this case, the congenital nature of the problem was evident, as the patient had an overall hypoplastic upper limb—the affected side was almost two cm shorter than the other side. Meticulous clinical and radiological assessment is required to diagnose this congenital deficiency and avoid unnecessary surgical intervention [8].

## Discussion

Our case report describes a bilateral absence of the lunate bone. The patient, in her early fifties, presented with pain during repetitive activities such as push-ups and routine office work.

Although the patient’s septic meningitis in early life could not be ruled out as a contributing factor, as proposed by Roche et al. the symmetry of the findings suggested a developmental anomaly.

In this case, the presence of an apparent ossification center that failed to develop into a mature lunate suggests that the underlying cause may be an interruption or arrest in normal ossification. A hereditary component seems unlikely, as the patient’s sister had only a trigger finger release and no other relevant abnormalities. The patient declined radiographic screening for her children, though such imaging could have provided additional insight into potential subclinical variants of carpal development. Furthermore, our findings could suggest a genetic explanation rather than a sporadic cause.

The patient’s symptoms improved following conservative therapy. Surgical intervention is not currently recommended. None of the reported cases in the literature have undergone surgery (similar to our case). Although synovectomy could theoretically be considered in select symptomatic cases, there are no well-established partial surgical options for this anatomical variant. Procedures such as partial wrist fusion or proximal row carpectomy are not suitable due to the undeveloped lunate fossa and associated hypoplasia of other carpal bones. Total wrist fusion is likely most reliable option as a operative treatment as the lunate fossa is probably also deformed and other carpal anomalies (hypoplastic scaphoid) are likely present.

In symptomatic or acquired conditions involving the lunate—such as advanced Kienböck’s disease—surgical options reported in the literature include lunate excision, various forms of carpal arthrodesis, prosthetic replacement, and vascularized bone grafting. However, the quality of evidence supporting the management of lunate absence remains low, consisting primarily of Level IV and V studies.

Due to limited sample size, single centre design and non-reproducibility of studies it is hard to draw strong conclusions. Despite these limitations, such case reports still provide valuable clinical insight, particularly regarding diagnostic challenges, anatomical variations, and appropriate treatment strategies for rare presentations like in our case of bilateral absence of the lunate. In view of the exceedingly limited literature of this rare entity, we suggest an algorithm that attempts to summarize the main details to be considered for treatment of absent lunate (Figure 4).

**Figure 4.**
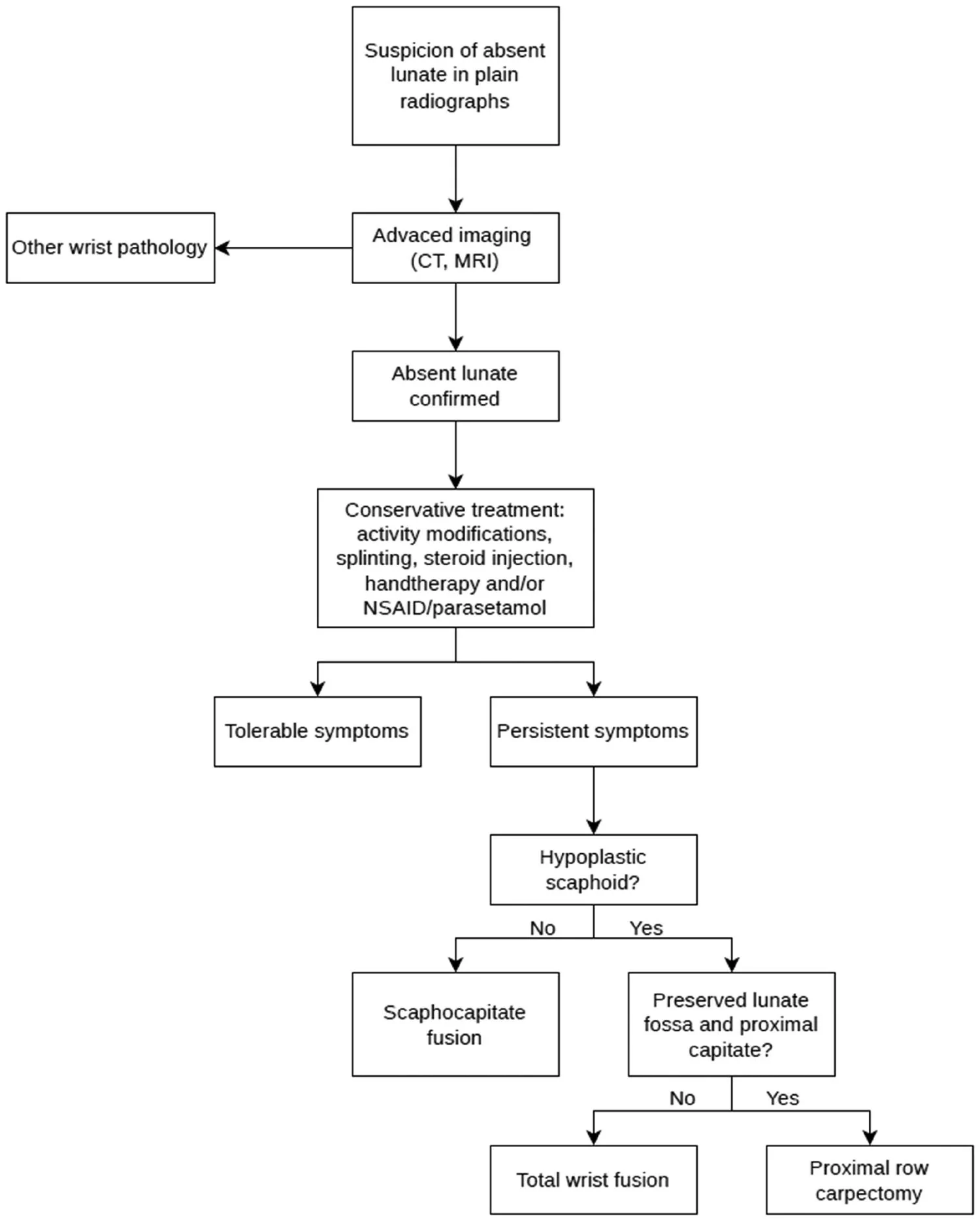
Diagnostic and treatment algorithm for suspected absent lunate.

## Conclusions

The absence of the lunate bone in adults is a rare clinical condition, most encountered as a congenital abnormality that causes limitations, likely due to secondary changes at a later stage. Diagnosis requires careful imaging and clinical correlation, and management should be tailored to the underlying cause and the severity of the patient’s symptoms. Although there are a limited number of surgical options, conservative measures should be favored if the symptoms are tolerable. Despite recent advances, there are still significant gaps in our understanding of the natural history, optimal management and long-term outcomes of adults with an absent lunate bone, particularly in congenital cases and following newer reconstructive techniques.

## Data avaliability statement

Data is contained within the article.

## References

[1] Dwivedi A, Singh P, Upadhyaya GK, et al. Congenital absence of lunate and triquetrum with hypoplastic scaphoid – a case report and review of literature. Int J Res Orthop. 2023;9(6):1279–1282. doi: 10.18203/issn.2455-4510.IntJResOrthop20233278.

[2] Butler P, Mitchell A, Healy JC. Applied radiological anatomy. Cambridge University Press; 2012. ISBN:0521766664

[3] Poznanski AK, Holt JF. The carpals in congenital malformation syndromes. Am J Roentgenol Radium Ther Nucl Med. 1971;112(3):443–459. PMID: 5570355. doi: 10.2214/ajr.112.3.443. 5570355

[4] Roche AF. Absence of the lunate. Am J Roentgenol Radium Ther Nucl Med. 1967;100(3):523–525. PMID: 4961620. doi: 10.2214/ajr.100.3.523. 4961620

[5] Kobayashi H, Kosakai Y, Usui M, et al. Bilateral deficiency of ossification of the lunate bone. A case report. J Bone Joint Surg Am. 1991;73(8):1255–1256. PMID: 1890129. doi: 10.2106/00004623-199173080-00017. 1890129

[6] Newcomb AH, Frankenkoff J. Unilateral congenital lunate absence: a case report. Sciedu Press. 2021;7(1):1–4.

[7] Edwin A, Bhardwaj P, Salyan S, et al. Isolated unilateral absence of lunate: a case report and review of literature. J Hand Surg Asian Pac Vol. 2024;29(3):252–255. Epub 2024 May 10. PMID: 38726494. doi: 10.1142/S2424835524720081. 38726494

[8] Timins M. Osseous anatomic variants of the wrist: findings on MR imaging. AJR Am J Roentgenol. 1999;173(2):339–344. doi: 10.2214/ajr.173.2.10430131. 10430131

